# Nerve in Surgery

**Published:** 1911-03-11

**Authors:** 


### The Hospital

A Journal of

The Medical Sciences and Hospital Administration.

New Series. No. 214, Vol. VIII. [No. 1286, Vol. XLIX] SATURDAY, MARCH 11, 1911

### Nerve in Surgery.

Nowadays it is a commonplace to assert that anæsthetics have, for the most part, discounted the necessity for speed, dexterity, and sureness of touch in the modern surgeon; that the days of the "chirurgeon," the man of his hands, are over; and that anyone with training can acquire sufficient manipulative dexterity for any ordinary form of operative procedure. The lightning dexterity of pre-anæsthetic days is no longer required; it is unnecessary now to be able to amputate the thigh in twelve seconds, the time credited to Fergusson. There is a water-colour drawing extant of Surgeon Macnamara, "primus et optimus," as the inscription has it, amputating the lower third of the thigh of a writhing sailor, who is being held down by six burly, blood-bespattered assistants. This little picture conveys in a manner untranslatable in words, the nerve, promptitude, and almost automatic dexterity required in the days when neither anæsthetics nor hæmostatic accessories were known. Happily such extreme rapidity is no longer required, but that the nerve necessary to undertake anything humanly possible is still with us, and that promptitude in an emergency is as great an essential as ever, no one acquainted with the recent trend of modern surgery can for a moment doubt.

The foregoing has been suggested by an extraordinary account in the *Presse Médicale* for February 11 of an operation for hernia performed on himself, under the influence of spinal anæsthesia, by a young Roumanian surgeon named Fzaicou, aged twenty-six. Suffering from a left inguinal hernia, an epiplocele the size of a hen's egg, which though readily reducible had become obstructed three times in the previous year, he decided it was necessary to have it operated on. Like most Continental surgeons he entertained an exaggerated idea of the risks of general anæsthesia, and so debated with himself whether he should have it done with local or spinal analgesia. As, however, he was preparing a thesis on Jonnesco's strychno-stovaine method, the daring thought occurred to him that, if it were possible to operate on himself, he would be able to describe, subjectively, all the symptoms in a manner that no amount of careful objective study could approach. This feat, in spite of the opposition of his colleagues and teachers, he attempted, as the result proved, with success, and a full description, with photographs taken during the operation, is contained in the paper.

He directed that 7 centigrammes of stovaine, and 1 milligramme of strychnine, injected between the eleventh and twelfth dorsal vertebræ, should be the dose and site. His colleagues, however, injected him in the usual lumbar site, and used only two-thirds of the specified dose. Sitting up on the operating table, with his legs hanging over the end, and his instruments collected all around him, he impatiently awaited the spread of the anæsthesia, and as he found it travelled very slowly, at the end of five minutes had himself lowered into the supine position. This caused the anæsthesia to spread into the inguinal regions; nevertheless, at the end of eighteen minutes after the injection, on attempting to make the skin incision he found he was still sensitive. His colleagues at this stage attempted to dissuade him from going on any further with the attempt, and refused to repeat the injection, as he requested, whereupon he injected himself locally in the groin with stovaine, which he had had prepared in case of such an emergency. Twenty-five minutes after the spinal injection he commenced the operation. It lasted an hour, the method performed was a modified Bassini, and there was no sensation of pain except when the cord was handled. Occasionally he felt a little vertigo when he stooped forward or turned his head rapidly. He was anæsthetic to the umbilicus, but no effort was produced on the manipulative skill of his fingers or on his intelligence, which remained perfectly clear.

Anæsthesia began to disappear about an hour after he was put back in bed. The temperature was above normal for a few days, and he had practically no sleep for the first three nights. Violent epigastric pain, relieved by pressure, was a constant feeling at first, besides pain in the back radiating from the spine, followed by frontal and temporal headache on the fifth day. At the end of a week all these symptoms had disappeared. From personal experience, therefore, he found that the anæsthesia came on and went off painlessly, was perfectly safe, could be limited in its upward spread by position, produced a practically complete loss of sensation in the parts operated upon, and did not interfere in any way with the reasoning faculties, or the fine movements of the hands necessary in performing the operation.

